# The Emerging Role of Visible Light in Melanocyte Biology and Skin Pigmentary Disorders: Friend or Foe?

**DOI:** 10.3390/jcm12237488

**Published:** 2023-12-04

**Authors:** Xuanxuan He, Shanglin Jin, Xiaoxi Dai, Li Chen, Leihong Xiang, Chengfeng Zhang

**Affiliations:** Department of Dermatology, Huashan Hospital, Fudan University, Shanghai 200040, China; hxx20000819@163.com (X.H.); jinshanglin203@126.com (S.J.); ynkmykdx123@163.com (X.D.); cl_1063@163.com (L.C.)

**Keywords:** visible light, pigmentary disorders, vitiligo, melasma, laser, LEDs, IPL

## Abstract

Electromagnetic radiation, notably visible light (VL), has complicated effects on human skin, particularly pigmentation, which have been largely overlooked. In this review, we discuss the photobiological mechanisms, pathological effects, clinical applications and therapeutic strategies of VL at varying wavelengths on melanocyte biology and skin pigmentary disorders. Different VL wavelengths may impose positive or negative effects, depending on their interactions with specific chromophores, photoaging, ROS production, circadian rhythm and other photon-mediated reactions. Further in vivo and in vitro studies are required to establish the pathologic mechanisms and application principles of VL in pigmentary disorders, as well as optimal photoprotection with coverage against VL wavelengths.

## 1. Introduction

**Figure 1:**
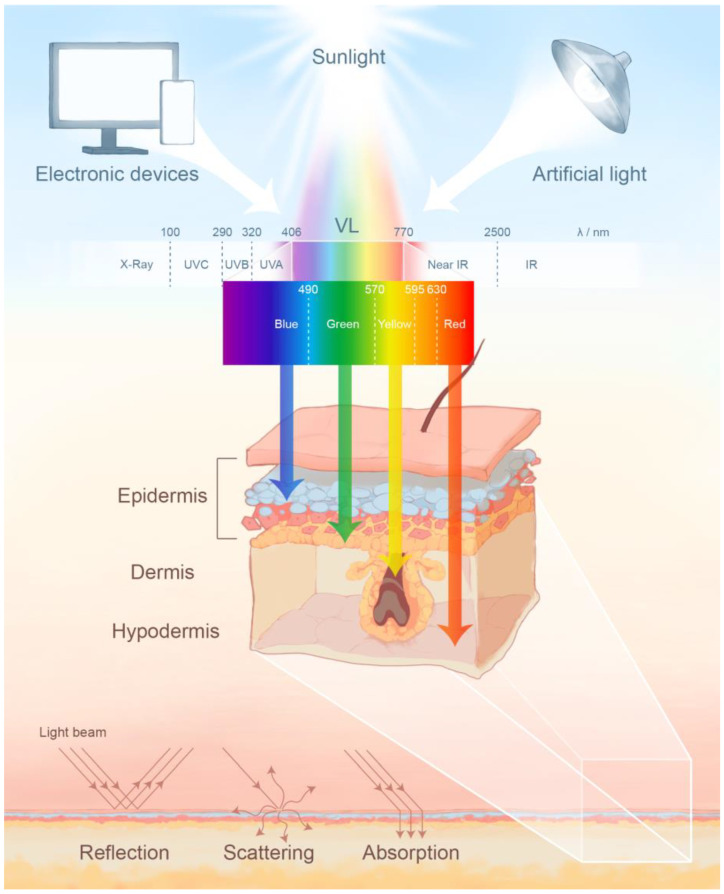
The classification, sources and penetration of visible light (VL).

Visible light (VL) refers to the narrow spectrum of electromagnetic radiation (EMR) that human eyes can perceive, with a wavelength range of 400 to 700 nm, although some individuals can also sense wavelengths from 380 to 780 nm [1]. Different wavelengths of VL cause various sensations to the human eyes, exhibiting different colors, which is also the principle of further division (Figure 1). Sunlight is the major source of VL, which makes up approximately 50% of sunlight encountering the Earth’s surface. Artificial sources of VL include flashlights, fluorescent lights, lasers, light-emitting diodes (LEDs), and other therapeutic devices [2]. In modern society, electronic devices such as computers and smartphones are also becoming increasingly prevalent as a source of radiation. Therefore, as the first barrier of the body, skin is bound to be affected by visible light exposure.

Skin pigmentary disorders, such as melasma and vitiligo, are characterized by hyperpigmented or depigmented lesions, and have a rapidly growing incidence worldwide. These disorders have serious impacts on people’s appearance as well as mental health [3], making it an urgent task to unveil the mystery of their pathogenesis and optimized treatment. While previous studies have mainly focused on the effect of ultraviolet (UV) radiation, the effect of VL on the skin, especially melanocyte biology, has been overlooked or considered negligible. The objective of this review is to enhance the understanding of the pathogenesis and management of pigmentary disorders by exploring the respective effects of VL at different wavelengths on melanocyte biology and diverse phototherapies for pigmentary disorders, using various light sources within the VL spectrum.

VL can be classified into primarily four categories, according to the wavelength and color: these are blue light, green light, yellow light and red light, with the wavelength of 400–490 nm, 490–570 nm, 570–595 nm, and 630–770 nm, respectively. The longer the wavelength, the deeper the VL cand penetrate into the skin. Therefore, red light can penetrate through the full thickness of the epidermis and dermis, reaching the subcutaneous layer, while blue light has less penetration. The sources of VL can be divided into three categories: natural light, artificial light and electronic devices. UV refers to ultraviolet; IR to infrared radiation.

## 2. The Photobiological Effects of Visible Light on Skin

It was previously believed that VL had minimal biological effects. However, a large number of researches in the past decades has made it evident that VL can exert significant impacts on various biological processes of skin, including chromophore activation, photoaging, oxidative stress, DNA damage and circadian rhythm, all of which may thus contribute more or less to skin pigmentation. It is to be note that different wavelengths of VL can have a synergistic or antagonistic effect on the same biological process.

### 2.1. The Activation of Chromophores by Visible Light

When a photon reaches the skin’s surface, it can be reflected, scattered, or absorbed (Figure 1), but only absorbed light can cause photobiological changes by interacting with chromophores [4]. Chromophores are photoreceptor molecules that can be activated and energized by photons to mediate biological effects [5]. Hemoglobin, cytochrome C oxidase (CCO), opsins (OPN) and melanin are the primary chromophores responsible for VL absorption, which is the premise and basis of the use of VL in laser therapy, intense pulsed therapy (IPL), and especially in low-level light therapy (LLLT) [6]. Chromophores absorb specific wavelengths of light, excite electrons to higher-energy states, and then activate second messengers such as ROS, Ca^2+^, ATP, cAMP, and NO, followed by the modulation of subsequent cascades of signaling pathways related to fundamental activities such as migration and proliferation, protein synthesis and tissue repair, inflammation, anti-apoptosis and redox reactions [7].

The relation between chromophore activation by VL and pigmentation in melanocytes has also been proved [8]. One study demonstrated that shorter wavelengths of VL (415 nm, 50 J/cm^2^) can activate OPN3, which senses blue light and then activates CAMKII, CREB, ERK and p38, thus up-regulating MITF signaling and inducing potent and sustained hyperpigmentation [9]. However, the activation may be dose-dependent, for another study showed that, under much smaller doses, blue light (450 nm, 200 mJ/cm^2^) and green light (550 nm, 200 mJ/cm^2^) failed to initiate human OPN3, which can act as a negative regulator of melanogensis when treated, by regulating the α-MSH–induced MC1R-mediated cAMP signaling [10]. Furthermore, Campiche et al. reported that LED blue light (450 nm, 60 J/cm^2^) resulted in changes in skin chromophores and signs of skin photoaging, including hyperpigmentation [11]. 

Different chromophores may exist in different layers of the skin. The depth of light radiation penetration is inversely proportional to the absorption rate and scattering rate, which are both inversely related to the wavelength [12]. Therefore, the longer the wavelength, the deeper the light can penetrate into the skin, meaning that VL can go even deeper than ultraviolet, and that VL in different wavelengths may activate chromophores present in different layers, resulting in diverse subsequent reactions.

Taken together, with deep penetration into the skin and multiple chromophore-mediated activities, VL has a profound and substantial impact on skin, including the epidermal melanin-unit system. 

### 2.2. The Regulation of Skin Aging by Visible Light

Skin photoaging is a complex process that involves degenerative changes in the skin, such as mottled hyperpigmentation and decreased elasticity and laxity caused by exposure to sunlight. This process is caused by the dysfunction of the nucleus, mitochondria, and the extracellular matrix (ECM) resulting from DNA damage and ROS generation [13]. 

Considering that VL participates in inflammation, oxidative stress, and the production of matrix metalloproteinases (MMPs), which is thought to be one of the crucial factors participated in ECM degradation in the skin [14], it is closely involved in the regulation of skin aging. However, different types of VL affect skin aging by distinct mechanisms, primarily targeting fibroblasts [15]. For instance, blue and green light have been proved to precipitate photoaging by increasing ROS and MMP-1, decreasing collagen type I in human fibroblasts [16,17,18,19] and inducing the production of singlet oxygen, followed by nuclear DNA damage in epithelial cells [20]. Besides inducing oxidative stress, blue light has also demonstrated the ability to damage fibroblast mitochondrial functionality [21]. In contrast, yellow light has been shown to protect against skin aging by increasing collagen I in the dermis [22] and upregulating the expression of antioxidant enzymes, to reduce the generation of UVA-induced MMP-1, phosphorylated stress-activated protein kinase (pJNK) and ROS, in human fibroblasts [23]. Similarly, low red light has been shown to stimulate fibroblast growth, upregulating antioxidant-related genes in human fibroblasts, reducing ROS, down-regulating MMP and reversing collagen I degradation of skin cells, exerting anti-aging effects [15,24,25,26].

As mentioned above, dermal fibroblasts, as the major skin cell implicated in VL-induced skin photoaging, have been shown to engage in the signal crosstalk between dermal and epithelial cells related to pigmentary disorders, acting on melanocytes by releasing abundant proteins, cytokines and growth factors [27]. Therefore, pigmentation is closely related to photoaging, and some pigmentary disorders, such as melasma, have been considered as photoaging disorders [28]. One study also inferred that photooxidation may be involved in the mechanism of pigmentation after blue-light irradiation [21]. Hence, it is essential to enhance the comprehension of the mechanisms through which yellow and red light can potentially impede the photoaging process, while blue and green light may give rise to exacerbation.

### 2.3. The Disruption of the Skin Circadian Rhythm by Visible Light

It is now well established that circadian oscillators exist not only in the suprachiasmatic nucleus (SCN) of the hypothalamus, but also in peripheral tissues, including the skin [29]. Studies have demonstrated that VL can disrupt the normal circadian rhythm, negatively affecting skin homeostasis. Exposure to blue and green light emitted from computer screens significantly suppresses and delays nocturnal melatonin secretion, disrupting the sleep–wakefulness cycle [30,31,32,33]. In addition, red-light (631 nm) exposure was shown to potentially delay the circadian clock and the onset of sleep, inducing circadian resetting, despite small samples [34]. VL also regulates the skin’s circadian rhythm by interacting directly with skin circadian clock genes, such as *PER1* and *BMAL1*, in peripheral pathways [35]. 

Circadian rhythm has intricate influences on physiological processes in the skin, including melanogenesis. Hardman et al. showed that silencing peripheral clock genes *BMAL1/PER1* in human skin and isolated melanocytes upregulated melanogenesis and thus increased melanin content [36]. A subsequent study further proved that the peripheral clock was crucial for well-organized melanin synthesis in normal melanocytes [37]. *BMAL1* also transcriptionally upregulates microphthalmia-associated transcription factor (MITF) and prevents UVB-induced DNA damage through enhanced melanin synthesis [38]. Since circadian disruption or altered clock genes can result in multiple skin diseases [39], considering its effect on melanin synthesis, it is highly possible that the circadian rhythm may participate in the process of skin pigment regulation, to some extent. And disruption of the circadian rhythm may accelerate skin aging, increase pigmentation and even induce pigmentary disorders [40].

## 3. Visible Light as an Enhancer of Skin Pigmentary Disorder

**Figure 2:**
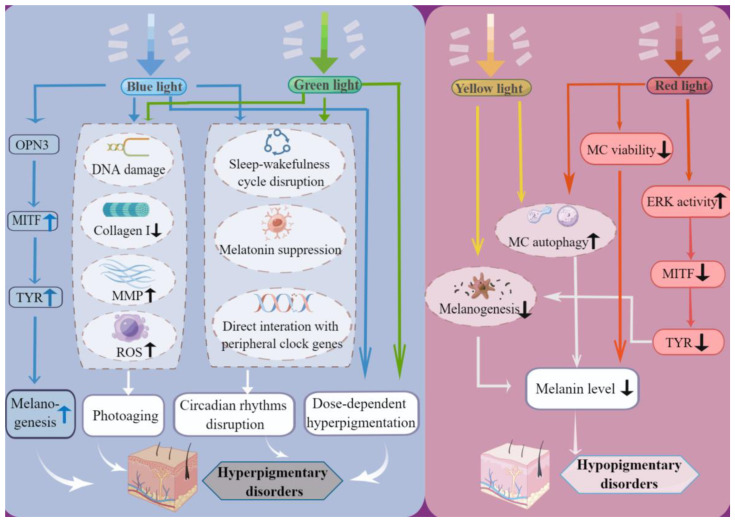
Pathologic effects of VL on pigmentary disorders.

Different regions of the VL spectrum are more likely to contribute to distinct pigmentary disorders. While blue or green light is generally considered one of the significant causes of disorders of hyperpigmentation under most conditions, in some cases, yellow or red light may serve as a crucial facilitator for the development of hypopigmentary diseases (Figure 2). 

Blue and green light may induce disorders of hyperpigmentation through several possible pathways: (a) direct interaction with OPN3; (b) photoaging aggravation; (c) circadian rhythm disruption; and (d) melanogensis upregulation. 

Yellow and red light impose possible stimulation on the onset and aggravation of vitiligo by affecting the viability and survival of melanocytes and the progression of melanin production. OPN3, opsin3; MITF, microphthalmia-associated transcription factor; TYR, tyrosinase; MC, melanocyte; ERK, extracellular regulated protein kinases; ROS, reactive oxygen species; MMP, matrix metalloproteinase; By figdraw.

### 3.1. Blue Light and Green Light Induce Hyperpigmentation

It is now well accepted that the short wavelength of VL plays a stimulatory role in hyperpigmentation. Multiple studies have shown that VL has synergistic effects with long-wavelength UVA1 on pigmentation [6,41,42]. VL-induced hyperpigmentation was found more potent and long-lasting than UVA1-induced hyperpigmentation in individuals with dark skins [6,43], even though the mechanism of hyperpigmentation caused by VL has been proved similar to that caused by UV [44]. Typically, three mechanisms are involved in the responsive reaction of melanocytes to VL, with increased melanin content: immediate pigment darkening (IPD), persistent pigment darkening (PPD), and delayed tanning (DT) [45]. IPD and PPD result from the oxidation of melanin precursors and the redistribution of melanin, while DT is associated with melanogenesis [46]. Randhawa et al. performed a series of ex vivo and clinical studies, and demonstrated that a single exposure to VL was ineffective in generating persistent pigmentation, while multiple exposure to VL induced PPD both in vivo and ex vivo [47]. In contrast, a classic study conducted by Mahmoud et al. demonstrated that a single dose of VL irradiation was enough to induce IPD and PPD in skin-type IV–VI individuals, and that higher doses (80–120 J/cm^2^) could induce DT [6]. Another study also revealed that both single high-dose (135 J/cm^2^) and repetitive (45 J/cm² over 5 consecutive days) exposure to blue light (450 nm) would result in IPD [48]. Those results indicate that VL, especially in the short wavelength, is able to induce apparent darkening via regulation of melanin redistribution, melanosome maturation or melanin synthesis. 

While numerous studies have documented that irradiation with blue light and green light can induce a dose-dependent hyperpigmentation response [6,11,43,45,49,50,51], the skin phototype plays a crucial role in this process. For instance, it has been proved that blue light (453 nm,18 J/cm^2^) can induce IPD in type I–III healthy skins [52], which is in line with the findings of Kleinpenning et al., who explored the clinical and histological effects of blue light (420 nm, 20 J/cm^2^) on normal skin types I–III. The authors observed transient melanogenesis and inexplicable vacuolization without melanocyte apoptosis [51]. Likewise, Moreiras et al. demonstrated that both blue light (450 nm) and green light (530 nm) induced melanin production in healthy human skin of types II and III ex vivo, without any detectable increase in DNA damage or cell apoptosis, even under fairly high doses of exposure (140 J/cm^2^) [53]. The same study evaluated melanin induction histologically in the epidermis of blue- and green-light-irradiated phototype I skin, although it was invisible, which is somewhat inconsistent with Mahmoud’s findings that melanogenesis induced by blue light (495 nm) and green light (595 nm) at 8–480 J/cm^2^ tends to occur in phototypes IV–VI skin, yet remains undetectable in lighter skin [6].

The clinical relevance of the capability of blue light and green light to induce pigmentation highlights the pathologic role of VL in photo-induced disorders of hyperpigmentation. It has been proposed that broad-spectrum sunscreens that do not adequately protect against VL fail to prevent worsening of post inflammatory hyperpigmentation (PIH) [54] and relapse of melasma [55,56]. A study involving 22 melasma patients showed that blue light induced melanogenesis both in the lesional and the neighboring skin, suggesting that blue light imposed a stimulatory effect on the onset and progression of melasma for the first time [57]. Although short-term exposure to blue light from electronic devices is not considered to exacerbate melasma [58], the low energy of artificial indoor VL is sufficient to induce hyperpigmentation in melasma patients [59]. Blue light emitted by the sun has also been proved to accelerate relapse of melasma [60].

### 3.2. Yellow Light and Red Light Induce or Aggravate Hypopigmentation

Yellow light inhibits melanogenesis and downregulates melanin content. In our former study, a dose-dependent inhibition of melanogenesis was observed, along with the induction of human epidermal melanocyte autophagy by yellow light irradiation (585 nm, 5–20 J/cm^2^) [61]. On the other hand, 630 nm red light (10–150 J/cm^2^) was found to have no promotion effect on melanogenesis in a study by Duteil L et al. [43], and turned out to impose an inhibitory effect on melanin synthesis, both in vitro and in vivo. Additionally, 633 nm (96 J/cm^2^) red light LED was reported to decrease melanin levels significantly after phototherapy of patients with acne vulgaris [62]. Similarly, 660 nm red light showed a depigmenting effect with downregulation of tyrosinase and MITF, due to increased ERK activity [63]. We previously revealed that red light (630 nm, 5–20 J/cm^2^) might decrease cell viability and increase apoptosis of melanocytes [61], which might also lead to hypopigmentation.

Limited clinical studies could be found on the promoting effect of VL on hypopigmentary diseases. However, several case reports showed that yellow light and red light may potentially induce depigmentation. Lee’s study showed that the melanin levels of the treated area in acne vulgaris patients slightly increased when treated with blue light (415 nm, 48 J/cm^2^) LED for 20 min, twice a week, for a duration of four weeks, while they significantly decreased when treated with red light (633 nm, 96 J/cm^2^) LED in the treated area [62]. What’s more, a 41-year-old woman who had no previous history of vitiligo or halo nevus developed vitiligo patches on the treatment site after IPL treatment for rejuvenation [64]. Moreover, using a 585 nm pulsed dye laser to treat port-wine stains was reported to induce depigmented patches and cause the Koebner phenomenon in vitiligo patients [65,66], something which has also been reported after 755 nm laser (red light) treatment [67,68].

### 3.3. Measures to Protect against Cutaneous Damage by Visible Light

#### 3.3.1. Exposure Reduction

As discussed previously, it is important to control light exposure, especially blue light from electronic devices and sunlight exposure. To prevent disruption of circadian rhythm by VL, reducing screen time by taking frequent breaks from long-period device usage and restricting artificial light at night are effective ways [69]. Simple but efficient physical methods for reducing sunlight exposure include seeking shade or staying indoors during peak hours, using a parasol and wearing photoprotective clothing and accessories, such as sunglasses or wide-brimmed hats [70], which have been proved to lower the chance of sunburn more significantly than photoprotection products on the market such as sunscreen and antioxidants [71]. While, theoretically, using standard window glass such as reflective or tinted automobile windows and window films that filter out VL may also help in reducing VL exposure, most glasses are mainly used for UV reflection, with little effect on VL protection [72,73], and are awaiting more exploration from the VL-protection perspective.

#### 3.3.2. Sunscreens

Over the past decade, sunscreens have undergone significant changes, due to the growing recognition that traditional broad-spectrum sunscreens with only organic or inorganic (ZnO and TiO_2_) UV filters do not provide adequate protection against VL [54]. Tinted sunscreens have emerged as a promising solution, which combines UV filters with different concentrations and ratios of iron oxides and titanium dioxide to protect against VL for individuals of all skin types. The critical component of tinted sunscreens is Fe_2_O_3_, which has three different colors, depending on its oxidation state: yellow, red, or black [70]. Among them, yellow Fe₂O₃ offers the strongest protection against VL-induced hyperpigmentation [74]. Studies have demonstrated that iron-oxide-containing tinted sunscreen significantly lessens the development of VL-induced hyperpigmentation [75] and that the efficiency increases to over 93% with increasing iron oxide content [76]. It has also been proved to benefit individuals with hyperpigmentation disorders such as melasma and PIH [60,77,78,79]. Additionally, Fe₂O₃-containing products with multiple shades and tones can be used to cover pigmentary blemishes [78], making them a novel cosmetic-friendly strategy for full-spectrum photoprotection beyond the UV range, and with a profound influence on patients with pigmentary disorders. 

#### 3.3.3. Antioxidants

It has been estimated that 50% of ROS generation can be attributed to VL and infrared radiation [80], which may induce melanogenesis [81] and exacerbate pre-existing hyperpigmentation [82]. Antioxidants can mitigate the harm caused by VL through ROS neutralization or melanogenesis pathway regulations [80] (Table 1).

WH130, a kind of licorice extract, inhibits melanogenesis by suppressing tyrosinase activity, particularly when heated, making it a promising option for treating various disorders of hyperpigmentation, including brown spots, ephelides, and melasma [83]. French maritime pine bark extract (PBE), with its antioxidant property, has also proved to reduce VL-induced melanin synthesis in vitro, via inhibiting tyrosinase and other pigmentation-related mediators [84]. Furthermore, a clinical study comparing antioxidant-enriched sunscreens with tinted sunscreens showed that the former had comparable or even better efficacy than the latter [85]. Another study also demonstrated that topical antioxidants inhibited erythema and reduced pigmentation caused by VL and UVA1, suggesting that antioxidants may prevent the exacerbation of pigmentary disorders due to sunlight exposure [86].

On the other hand, multiple studies have provided theoretical evidence that antioxidants may attenuate hyperpigmentation by reducing oxidative damage and preventing VL-triggered photoaging. Sunscreens containing antioxidants have been shown to repair some clinical signs of photoaging [87]. Hydroxytyrosol from olive fruits prevents human keratinocytes and fibroblasts from blue-light-induced photoaging [18], while resveratrol can potently scavenge ROS induced by blue light (415 nm) in fibroblasts [16]. Licochalcone A, a Nrf2 inducer, has been reported to reduce ROS formation in vitro and prevent intradermal carotenoid depletion in vivo [19]. Furthermore, polypodium leucotomos extract (PLE) may also offer protection against VL-induced photoaging. PLE treatment led to a significant decrease in VL-induced PPD and DT and a reduction in the markers for cellular damage [88]. PLE was also reported to prevented human dermal fibroblast damage and mitigate photoaging-related ECM degradation in vivo by reducing VL-induced MMP-1 [89,90]. Relatively, scientific and commercially available antioxidants against UV radiation are much more abundant than those against VL [91]. Therefore, the potential of antioxidants targeting VL remains to be extensively explored.

**Table 1:** The hyperpigmentation attenuated functions of antioxidants targeting VL.

Antioxidants	Mechanisms	Origins	Objects
WH130	Inhibits melanogenesis by suppressing tyrosinase activity	Extract from Licorice (Wongam);	Murine melanoma B16F10 cells [83]
PBE	Reduces VL-induced melanin synthesis by reducing tyrosinase activity and decreasing ED1, and PPAR α, δ, and γ production	Extract from French maritime pine bark (*Pinus pinaster*)	Human melanocytes [84]
Resveratrol	Scavenges ROS induced by blue light (415 nm) LED in human fibroblast	Root extract from *Veratrum grandiflorum*	Human Skin Fibroblasts [16]
Hydroxytyrosol	Protects keratinocytes and fibroblasts from damage induced by blue light through preventing ROS formation, reducing MMP levels, preserving collagen type I production, and decreasing DNA damage	Extract from olive fruits	Human keratinocytes and fibroblasts [18]
Licochalcone A	Decreases VL-induced ROS formation in human fibroblast to a level equivalent to unirradiated fibroblast cells, or even below, in vitro, and prevents intradermal carotenoid depletion by VL irradiation in vivo	Root extract from Licorice (*Glycyrrhiza inflata*);	Human dermal fibroblasts and 10 healthy subjects with Fitzpatrick skin phototype II or III [19]
PLE	1. Decreases PPD and DT; 2. Decreases cyclooxygenase-2 and cell damage; 3. Prevents alterations in morphology, cell survival and cell cycle of human dermal fibroblasts and changes in the expression of MMP-1, CTSK, fibrillins 1 and 2 and elastin, caused by VL	Extract from *Polypodium leucotomos;*	22 subjects with Fitzpatrick skin phototype IV–VI [88] Human dermal fibroblasts [90] 7 healthy subjects [89]
Carotenoid	Filters out high-energy blue-light rays	Diets	46 healthy subjects [92]
Flavonoid	Decreases photosensitivity of phospholipids to blue-light oxidative damage	Extract from green tea	Langmuir monolayers of 1,2-dipalmitoyl-sn-glycero-3-[phospho-rac-(1-glycerol) (sodium salt) (DPPG) [93]
Vitachelox	Protects human keratinocytes by reducing oxidative damage (protein carbonylation) induced by blue-light radiation.	A mixture of three natural extracts: grape (*Vitis vinifera*) seeds, green tea (*Camellia sinensis* green) leaves, and oak (*Quercus robur*)	Human keratinocytes [94]

## 4. Visible Light as a Therapeutic Option for Pigmentary Disorders

Visible-light therapy (VLT) is commonly used for various skin diseases, mostly as a second-line option. Likewise, it plays a primary or adjunctive role in the clinical management of pigmentary disorders. In the treatment of pigmentary disorders, there are three primary types of visible-light therapies utilized: laser, IPL, and LED therapy. Each type of light has unique features and mechanisms that cater to different skin conditions and disorders (Table 2).

### 4.1. Laser-Emitting Lights in the Visible Range

Visible lasers are a class of laser devices that emit light in the visible-spectrum region, containing pulsed dye laser (PDL), copper vapor laser, potassium titanyl phosphate laser (KTP), helium-neon (He-Ne), ruby laser, argon laser and krypton laser [95]. As a coherent light, laser has the advantages of high intensity, low divergence, and precise control over the amount and location of skin heating [96], which makes it an ideal method for treating skin diseases based on the principle of selective photothermalmolysis [97].

While PDL was initially designed for cutaneous vascular disorders, recent studies have shown that 595 nm and 607 nm PDL can also be used to treat benign epidermal pigmented lesions (EPLs) [98,99,100,101,102,103,104]. And 585 nm and 595 nm PDL have also been found to be effective in improving melasma lesions that exhibit increased vascularity, with or without the combination of other therapies [105,106,107].

Another type of laser that is highly specific for vascular lesions is copper vapor laser, emitting a dual wavelength comprising 10% 511 nm and 90% 578 nm, which is at the proximity of the absorption peak of hemoglobin [108]. Nonetheless, the dual-wavelength copper vapor laser shows great efficacy in eliminating congenital melanocytic nevi (CMN) [109], yet demonstrates less efficacy in treating melasma patients [108,110], except for those with pronounced vascular abnormality [111].

The KTP laser, also known as the (Q-switched) Nd:YAG double-frequency 532 nm laser, is another type of laser that has been proved effective in treating EPLs, such as ephelides or solar lentigines [112,113,114,115], physiological lip hyperpigmentation (PLH) [116,117], and even tattoos [118,119]. When combined with IPL, it has been successful in treating postoperative inflammatory hyperpigmentation [115].

The 633 nm He-Ne laser emitting red light is a popular choice for low-level light therapy (LLLT), and has been found to be effective for vitiligo. Yu et al. discovered that low-energy He-Ne lasers (632.8 nm) enhance melanocyte migration and proliferation, and even rescue damaged melanocytes, creating a positive microenvironment for repigmentation [120]. The same group also investigated the molecular mechanism and biological effects of the low-energy He-Ne laser on pigment cells at different maturation stages. They found that the laser induced differentiation and mitochondrial biogenesis of primitive pigment cells through calcium-dependent mitochondrial retrograde signaling [121], as well as stimulating the differentiation of immature melanoblasts through enhanced pp125FAK expression and the melanogenesis of more mature melanoblasts [122]. Furthermore, they explored the role of the low-energy He-Ne laser in melanocytes, and demonstrated enhanced functional melanocyte proliferation via increased expression of ɑ2β1 integrin and increased attachment to collagen IV [123]. These studies provide a solid theoretical basis for understanding how low-level laser therapy induces repigmentation in vitiligo. Clinical evidence also supports the application the of low-energy He–Ne laser in treating segmental-type vitiligo, with an effectiveness comparable to conventional therapies [120].

Interestingly, red light can also be used as an effective and safe modality for further depigmentation of vitiligo. The cosmetically disturbing remnants of normal pigmentation in patients with vitiligo whose skin has been almost depigmented on the whole can be removed by the Q-switched 694 nm Ruby laser (QSRL) [124]. The QSRL is particularly effective for treating benign pigmented diseases, such as tattoos, nevus of Ota and ephelides, due to its high absorption by melanin [125,126,127,128,129,130]. While QSRL was previously believed to be ineffective in treating melasma [131], recent studies with small sample sizes have demonstrated its efficacy [132,133,134]. Similarly, the Q-switched 755 nm Alexandrite laser (QSAL) can also be used to treat a variety of superficial and deep hyperpigmented diseases such as nevus of Ota/Ito, tattoos, café au lait macules and melasma [129,135,136,137]. QSRL and QSAL are considered the best choices as phototherapy for treating dermal pigmented lesions by Bogdan et al. [102]. Yet the newly developed picosecond laser with higher efficiency in pigment removal and less thermal damage is worthy of consideration [138,139].

Taken together, the visible laser can attenuate or eliminate hyperpigmentation to a certain extent, except for the He-Ne laser, which is usually used for depigmentation. Among them, the PDL and copper vapor laser are classic modalities for vascular lesions, with recently discovered use in benign EPLs and melasma with a vascular component. KTP can tackle both epidermal and dermal hyperpigmentation, with a better effect on the former. QSRL and QSAL are best applied in dermal hyperpigmentation, but also have solid efficacy on EPLs, with new findings relating to melasma treatment that may renew the conventional views.

### 4.2. Intense Pulsed Light (IPL)

IPL is a polychromatic and noncoherent light released by a high-energy tritium flashlamp under high voltage, featuring high intensity, a relatively concentrated wavelength, and a wide and tunable pulse width. The IPL spectrum primarily falls in the range of 500–1200 nm, and can be selectively filtered by filters based on the skin type and lesions. IPL also works based on the principles of selective photothermalmolysis effects, as does laser [140]. Clinical studies have demonstrated that IPL is capable of effectively decreasing melanin production and accumulation at the cellular level, making it a suitable treatment option for various types of hyperpigmented skin conditions [141].

IPL has been proven effective in treating lentigines, ephelides, poikiloderma of Civatte and other epidermal hyperpigmentation, as well as benign melanocytic nevi such as Becker’s nevus [98,142,143,144,145,146,147]. However, it should be noted that the Q-switched laser still remains the preferred choice in light therapy for treating benign pigmented lesions. Furthermore, IPL is not a viable solution for tattoo removal, as it lacks the ability to perform Q-switching in incoherent light sources [148].

In the treatment of melasma, IPL has demonstrated superior efficacy when compared to laser treatment. In a split-face comparative study conducted by Hassan et al., IPL was observed to more effectively lighten epidermal melasma and melasma lesions with vascular alteration, in comparison to PDL [106]. Li et al. also demonstrated that IPL was ideal for melasma treatment with minimal and acceptable adverse events [149], which is consistence with Yi’s conclusion [150]. In addition to skin brightening, IPL has been popularly employed for skin rejuvenation, owing to its remarkable efficacy in addressing photoaging concerns [151].

### 4.3. Light-Emitting Diodes (LEDs)

**Figure 3:**
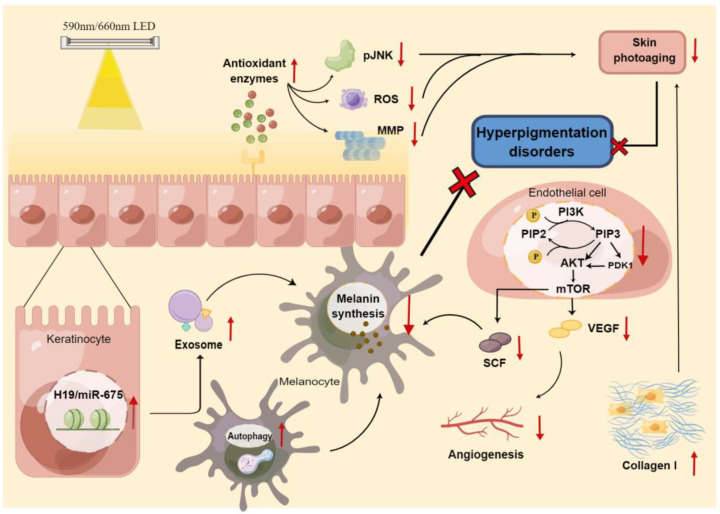
The mechanism of LED phototherapy for hyperpigmentation disorders.

LEDs emit incoherent light with a narrow spectrum and low intensity, which induces a mild effect on cells for regulating biological activity, rather than a thermal or exfoliative effect. This process is referred to as photomodulation or photobiomodulation (PBM), also known as LLLT [152]. A vast array of LED semiconductor materials has been available at lower wavelengths, and research over the past decade suggests that LED therapy is more suitable than laser therapy for LLLT, due to its mild output and convenient accessibility [153]. LED therapy using VL for pigmentary disorders has been a controversial approach, but recent studies have shed light on its potential application in melasma (Figure 3).

As previously mentioned, 585 nm yellow LED light has been showed to inhibit melanogenesis in melanocytes by the inducing of autophagy [61]. This was further explored by our later study, which demonstrated that irradiation with 585 nm LEDs resulted in the containment of melanin synthesis by upregulating H19 and its exosomal miR-675 derived from keratinocytes in vitro [154]. Our subsequent study further demonstrated in vivo and in vitro that 590 nm yellow LED decreased the secretion of melanogenic factor and reduced the angiogenesis of the human microvascular endothelial cell (HMEC-1) by dampening the PI3K/AKT/mTOR signaling pathway, thus prominently attenuating erythema and hyperpigmentation in melasma [155]. This series of experimental data is consistent with Mpofana’s study, in which 633 nm-LED combined with 830 nm-LED exposure significantly ameliorated melasma in patients with skin types V and VI [156]. In addition, our recent clinical trial focusing on melasma patients who underwent 590 nm LED treatment further proved that home-based 590 nm LEDs exhibited a similar efficacy and safety as in-hospital 1064 nm QSNY, with higher portability and lower cost [157].

From another point of view, phototherapy using LED to treat skin photoaging is increasingly prevalent. Lee et al. conducted a prospective split-face clinical study on LED phototherapy for skin rejuvenation, and indicated an altered enzymatic activity related to dermal matrix remodeling, as well as a reduction in melanin content after irradiation with 633 nm red LED [158]. Moreover, 590 nm-LED or 660 nm-LED therapy increased collagen and decreased MMP-1 activity in the dermis, with pigmentation reduction [22,23,25,159], suggesting a novel perspective for LED therapy to tackle melasma, which is now defined as a photoaging disorder [28].

While yellow and red LEDs are promising for treating disorders of hyperpigmentation, LED blue light has been applied for vitiligo repigmentation. Research indicates that blue LED, combined with Buddleja officinalis, can be used to treat vitiligo through induced melanin production by promoting melanogenic signaling, in addition to CREB/MITF/TYR pathways [160]. A retrospective study also demonstrated that 417 nm blue LED induced repigmentation in 30 patients with localized vitiligo, of varying ages and different skin types [161]. Despite the relatively small sample, these results encouraged the utilization of LED on melanin-deficiency skin diseases.

Of note, LED treatments have the special advantages of high safety and convenience with fewer side effects, yet well-designed studies with larger sample sizes and repeated measures of response are sorely lacking and highly required.

LED can be applied in disorders of hyperpigmentation treatment by directly affecting melanin production through various pathways or by alleviating the photoaging process, including antioxidant enzyme and collagen I production. Red cross refers to inhibition; Red arrows refer to upregulation or downregulation. ROS, reactive oxygen species; MMP, matrix metalloproteinase; PIP, phosphatidylinositol phosphate; PI3K, phosphatidylinositol 3-kinase; mTOR, mammalian target of rapamycin; SCF, stem cell factor; VEGF, vascular endothelial growth factor; By figdraw.

## 5. Perspectives

The threat posed by hyperpigmented or depigmented lesions to patients and society as a whole prompts deeper and more extensive research from angles beyond the traditional etiology and therapeutics. VL has thus emerged as a rapidly evolving field in photomedicine, and here we collated and clarified the detrimental and beneficial impacts of VL on melanocyte biology and pigmentary disorders, hoping to better instruct the prevention and treatment strategies of refractory pigmentary disorders in clinical practice. Despite this, studies attempting to determine the effects of VL on skin pigmentary disorders are still woefully inadequate and require further exploration, on multiple levels.

Firstly, the harmful effects of VL, including skin photoaging and circadian disruption, highlight the importance of proper protection against VL. As the evidence continues to mount, additional research on VL photoprotection is needed for sunscreens and antioxidants. Moreover, greater attention must be paid during medical treatments to take into account intensity, dose, exposed area, exposure duration, expose frequency, operation mode, and skin phototype-dependent differences in the pigmentary response of VL.

Secondly, despite some limited clinical evidence regarding the inducement of vitiligo by red or yellow light, the exact pathologic role of VL in depigmenting diseases has not been clearly determined. More fundamental and clinical studies are needed to clarify the precise role of VL in disorders of hypopigmentation.

Thirdly, in the last 5–8 years, the progress and development of pigment removal using QSRL has been slow, due to the lack of clinical and in vivo research. It is crucial to conduct studies with larger samples and more-strictly conducted procedures, to confirm the effectiveness of QSRL on melasma.

Last but not least, it is worth noting that LED, particularly yellow light, has exhibited great potential for melasma treatment, with the emerging fundamental and clinical studies. Considering its portability and economic applicability, LED yellow light is highly promising as a therapeutic alternative for treating melasma. There is a need for further clinical research, though, to determine the specific benefits of LED in treating other pigmentary disorders besides melasma, and to thus open up new fields of investigation and markets for both skin darkening and skin lightening.

In conclusion, like a double-edged sword, VL plays distinct roles in the onset, progression, and treatment in skin pigmentary disorders under different parameters and modes, targeting different skin-phototype individuals. More basic and clinical studies are merited to explore the precise mechanisms of pigment metabolism through VL regulation, which may provide a scientific basis for more effective prevention and management of photo-aggravated pigmentary disorders.

## Figures and Tables

**Table 2:** Differences between LED, Laser and IPL.

	Laser	IPL	LED
Intensity	High	High	Low
Pulse width	Unadjustable	Continuous and adjustable	Continuous and adjustable
Coherence	Coherent	Incoherent	Incoherent
Directionality	High/Single	Low/Multiple	Low/Multiple
Wavelength and Chromaticity	Monochromatic	500–1200 nm, Polychromatic	400–800 nm Polychromatic
Spot Size	Small point: <1 cm × 1 cm	Medium: 5 cm × 2 cm	Large: 30 cm × 30 cm
Mechanism	Selective Photothermalmolysis	Selective Photothermalmolysis	Photobiomodulation
Indications for pigmentary disorders	Benign epidermal pigmented lesions (ephelides, lentigo, PLH, café au lait macules, pigmented seborrheic keratoses…); Benign dermal pigmented lesions (CMN, nevus of Ota/Ito…); Mixed (epidermal/ dermal) pigmented lesions (Becker’s nevus, melasma, PIH); Tattoos; Vitiligo	Benign epidermal pigmented lesions (ephelides, lentigo, café au lait macules, …); mixed (epidermal/dermal) pigmented lesions (Becker’s nevus, melasma, PIH, poikiloderma of Civatte)	Melasma; Vitiligo
Adverse effects	Relatively common, mainly hyperpigmentation, sometimes scarring	Infrequent, sometimes erythema and hyperpigmentation	Rarely seen
